# Preconception care utilisation among women of reproductive age in Ghana: using Andersen's behavioural model

**DOI:** 10.3389/frph.2026.1874979

**Published:** 2026-08-05

**Authors:** Amidu Alhassan, Boahemaa Adu Otchere, Godswill Sedinam Lanyo, Frederick Acheampong Nimo, Mildred Awinpiini Asore, Justice Enock Kagbo, Bernard Nabe, Edward Odoom, William Akoto-Buabeng, Jerry John Ouner, Mustapha Amoadu, Patience Fakornam Doe

**Affiliations:** 1 Department of Adult Health, School of Nursing and Midwifery, College of Health and Allied Sciences, University of Cape Coast, Cape Coast, Ghana; 2 Department of Education and Psychology, University of Cape Coast, Cape Coast, Ghana; 3 Department of Family Health Care Nursing, School of Nursing, University of California, San Francisco, San Francisco, CA, United States; 4 Biomedical and Clinical Research Centre, College of Health and Allied Sciences, University of Cape Coast, Cape Coast, Ghana; 5 Department of Public Health, School of Nursing and Midwifery, College of Health and Allied Sciences, University of Cape Coast, Cape Coast, Ghana

**Keywords:** andersen's behavioural model, determinants, Ghana, preconception care, reproductive health, utilisation

## Abstract

**Background:**

Preconception care is an essential reproductive health intervention aimed at reducing preventable maternal and neonatal risks. Despite its significance, utilisation in low- and middle-income settings remains low. This study examined determinants of PCC utilisation among women of reproductive age in Ghana, applying Andersen's Behavioural Model.

**Methods:**

A descriptive cross-sectional design was used, involving 464 women aged 15–49 years in the Koforidua Municipality. Data were collected through structured questionnaires and analysed with descriptive statistics, Chi-square tests, and hierarchical logistic regression using Jamovi version 2.7.23.

**Results:**

The study found a PCC utilisation rate of 69.4%. Younger women were less likely to use PCC, with those aged 21–25 years (AOR = 0.03; *p* = 0.024) to 36–40 years (AOR = 0.01; *p* = 0.005) showing lower odds compared with women under 20 years. Ethnicity strongly influenced utilisation, with Akan (AOR = 6.94; *p* < 0.001), Ewe (AOR = 5.65; *p* = 0.007), and Ga (AOR = 5.10; *p* = 0.048) women having higher odds. Women with no formal education were less likely to utilise PCC (AOR = 0.09; *p* = 0.049), while those receiving counselling (AOR = 5.15; *p* < 0.001) or exposed to radio campaigns (AOR = 3.30; *p* = 0.003) had increased odds.

**Conclusion:**

PCC utilisation in Ghana was moderate and associated with sociodemographic, informational, and health-system factors. Strengthening provider counselling, community-based health education, and consideration of PCC within national reproductive health financing strategies may help improve uptake.

## Introduction

Preconception care (PCC) refers to evidence-based biomedical, behavioural, and social interventions delivered before conception to reduce maternal and neonatal risks and improve pregnancy outcomes (1). Globally, an estimated 260,000 maternal deaths occurred, equivalent to 700 deaths each day (2). Neonatal deaths are also alarming, with nearly 2.3 million newborns dying within the first 28 days of life in 2023 (3). PCC is an essential preventive measure for improving reproductive health outcomes through early risk identification and modification before conception (4, 5).

Across sub-Saharan Africa (SSA), utilisation of PCC remains low despite its proven benefits (6–8). Woldeyohannes et al. (9) found that only about one-quarter of women of reproductive age utilise PCC services. Gebretsadik et al. (10) reported high levels of preconception health risks among African women, including anaemia (36.7–58.1%), overweight or obesity (8.3–76.7%), unintended pregnancy (4.2–94.3%), sexually transmitted infections (1.3–29.2%), and intimate partner violence (6.7–43.7%) (10). These risks underscore the critical need for strengthening PCC interventions as a preventive approach.

In Ghana, neonatal mortality remains at 28 deaths per 1,000 live births (11), while completion of at least eight antenatal contacts is still low (12), reflecting missed opportunities for early intervention. Also, preconception risk factors remain prevalent in Ghana, with studies reporting that approximately 38.9% of pregnancies are unintended, while alcohol use among women of reproductive age is common and significantly associated with unhealthy pre-pregnancy behaviours (10, 13). Facility-based studies have shown limited awareness and service provision (14–16). Bawah et al. (16) reported that midwives in Northern Ghana identified inadequate resources, training, and time constraints as barriers to PCC delivery. Preconception Health Care (PCH) risks are high among Ghanaian women. However, women had a higher risk of anaemia, with nonpregnant and pregnant women experiencing 23% and 43% increased risk, respectively (17, 18). Alcohol use (37.8%–48%) (19, 20) and low PCC uptake worsens maternal–neonatal outcomes (21, 22). Similarly, Adjei et al. (23) found that women who accessed PCC had improved pregnancy outcomes compared with those who did not.

A complex interaction of sociodemographic, health-system, and behavioural factors influences utilisation of PCC. Andersen's Behavioural Model (ABM) categorises these into predisposing, enabling, and need-related factors (24). According to the model, health service utilisation is influenced by personal attributes like age and beliefs, resources, access, such as income, distance, and perceived or actual health requirements that drive care-seeking. In the current study, predisposing factors such as age, marital status, educational attainment, and reproductive history determine individuals' likelihood to seek care. Women with higher education and prior adverse pregnancy outcomes are more likely to utilise PCC (9). Enabling factors, including health insurance coverage, access to healthcare facilities, and exposure to health information, play a pivotal role (10). Additionally, perceived need for care, self-efficacy, and awareness of PCC benefits influence decision-making. Cultural beliefs, gender roles, and partner support also affect uptake, particularly in patriarchal societies where male consent may influence reproductive decisions (23). Addressing these determinants is vital to improving equitable access and utilisation across populations.

Despite global recognition of PCC as a vital reproductive health strategy, evidence explaining the determinants of PCC utilisation in Ghana remains limited, particularly within community and municipal settings. Most existing studies are hospital-based (15, 23, 25), focused mainly on awareness and knowledge, and limited in their ability to explain utilisation patterns across broader populations. In addition, few studies have applied Andersen's Behavioural Model to examine how predisposing, enabling, and need factors influence PCC utilisation among women of reproductive age. This evidence gap limits the development of context-specific reproductive health interventions and may contribute to continued exposure to preventable preconception risks, inadequate counselling, and poor pregnancy outcomes. Therefore, this study applied Andersen's Behavioural Model to examine determinants of PCC utilisation among women of reproductive age in the Koforidua Municipality. The findings are expected to provide evidence to inform local reproductive health planning, strengthen counselling interventions, promote equitable access to PCC services, and support progress towards Sustainable Development Goal 3, particularly target 3.7, which advocates universal access to sexual and reproductive healthcare services (26).

## Methods

### Design and population

This study adopted a descriptive cross-sectional design. This design was appropriate as it enabled the collection of quantitative data at a single point in time, facilitating the identification of associations between variables. The study population comprised women of reproductive age (15–49 years) residing in the Koforidua Municipality. This population was selected because it is the target group recommended by the WHO to receive PCC. A multi-stage sampling approach was employed. Healthcare facilities in the Koforidua Municipality were first stratified into primary, secondary, and tertiary levels to ensure representation across levels of care. Facilities were then purposively selected based on the provision of reproductive and maternal health services, including PCC-related care. Eligible women attending these facilities during data collection were consecutively recruited using convenience sampling after meeting the inclusion criteria and providing consent. Although convenience sampling may introduce selection bias, inclusion of participants across multiple facility levels enhanced diversity and minimised underrepresentation of key subgroups. The sample size of 478 participants was determined using a 95% confidence level, 5% margin of error, and a 25% non-response adjustment to ensure statistical robustness and adequate power for analysis.

The study included women of childbearing age (15–49 years) who had resided within the Koforidua Municipality for at least six months and were either planning to conceive, currently pregnant, or had recently given birth, irrespective of their pregnancy intentions. Women who had serious and acute health conditions requiring urgent medical attention or those with diagnosed mental health conditions were excluded to prevent bias arising from impaired participation or health-related limitations that could affect their ability to provide accurate and informed responses.

### Measures

The study utilised a structured survey questionnaire adapted from validated instruments on PCC awareness (6, 27–30). This study adapted an instrument based on items from the Health Literacy Scale for Preconception Care (31), and has been used in several empirical studies that assessed participants' knowledge of preconception health (28, 32–35). The instrument comprised two main components, the Behaviour and Skills Scale (25 items across six factors) and the Knowledge Scale (13 items). The Knowledge Scale included 13 multiple-choice questions designed to evaluate individuals' understanding of essential preconception health topics such as reproductive life planning, micronutrient supplementation, risk assessment, and counselling. An example of an item is, “Which of the following is incorrect about pregnancy?” Although the Knowledge Scale demonstrated a slightly lower Cronbach's alpha coefficient of 0.661, it was considered an acceptable measure for our study (0.865) for assessing knowledge-based competencies. The questionnaire was translated into Twi for those who cannot read and back-translated for linguistic accuracy. Pretesting with 20 women ensured cultural appropriateness, and Cronbach's alpha value above 0.825 confirmed internal consistency.

The dependent variable was utilisation of PCC, coded as “Yes” or “No”. Explanatory variables were selected based on Andersen's Behavioural Model. Predisposing factors included age, marital status, ethnicity, education, employment, religion, and household size. Need-related factors comprised pregnancy history, antenatal care attendance, pregnancy complications, contraceptive use, reproductive health awareness, and counselling from health workers. Enabling factors included income, health insurance, facility accessibility, privacy and confidentiality, NHIS coverage, distance to facilities, and exposure to radio campaigns, as these influence access to and utilisation of preventive reproductive healthcare services.

### Data collection and ethics

The study was conducted according to the Declaration of Helsinki. Data collection was conducted over a period of six to eight weeks from August 2025 to September 2025 by trained research assistants who ensured precision, confidentiality, and ethical compliance throughout the process. Ethical clearance was obtained from the Ghana Health Service Ethics Review Committee (GHS-REC:019/06/25), and an introductory letter from the Department of Adult Health, University of Cape Coast, was secured to facilitate access to the study sites. Before participation, respondents were fully briefed on the study's objectives, procedures, potential benefits, and voluntary nature, after which written informed consent was obtained. Privacy and anonymity were strictly maintained, with participants assured that the information collected would be used solely for academic purposes. Furthermore, participants were informed of their right to withdraw from the study at any point without facing any form of disadvantage or penalty.

### Data analysis

Data from the completed questionnaires were entered directly into a Google Form, which automatically generated an Excel dataset for analysis. Double-entry validation and consistency checks were performed to ensure data accuracy and completeness. PCC utilisation was coded as a binary outcome variable, where “Yes” was assigned a value of 1 and “No” a value of 0. The dataset was analysed using Jamovi version 2.7.23. Descriptive statistics and Chi-square tests were used to summarise the data and examine associations between explanatory variables and PCC utilisation. Binary logistic regression analysis was then conducted using a sequential blockwise modelling approach based on ABMHSU. Predisposing factors were entered in Model 1, need-related factors in Model 2, and enabling factors in Model 3 to assess their contribution to PCC utilisation. Findings were presented as adjusted odds ratios (AORs) with 95% confidence intervals (CIs).

## Results

### Sociodemographic characteristics of respondents

The study achieved a response rate of 98%. The mean age of respondents was 29.4 years (SD = 6.57), with most participants aged 26–30 years (31.0%) and 31–35 years (22.0%). A large proportion were single (52.8%), while 44.8% were married. The Akan ethnic group constituted the majority (53.9%), followed by Ewe (19.6%) and Ga (10.1%) women. Educationally, nearly half (46.6%) had a tertiary education, and 31.7% had completed senior high school. In terms of employment, 56.3% were employed, while 43.8% were unemployed. Christianity was the predominant religion (90.9%), with a smaller proportion identifying as Muslim (8.8%) or belonging to other faiths (0.2%) (Table 1).

**Table 1:** Sociodemographic characteristics of respondents (*N* = 464).

Variable	Sub-category	Frequency (*N*)	Percentage (%)	Mean	SD
Age (Years)				29.4	6.57
	Less than 20 years	35	7.5%		
	21–25 years	76	16.4%		
	26–30 years	144	31.0%		
	31–35 years	102	22.0%		
	36–40 years	70	15.1%		
	41–45 years	29	6.3%		
	More than 46 years	8	1.7%		
Marital status	Divorced	7	1.5%		
	Married	208	44.8%		
	Single	245	52.8%		
	Widowed	4	0.9%		
Ethnicity	Akan	250	53.9%		
	Dagomba	20	4.3%		
	Ewe	91	19.6%		
	Ga	47	10.1%		
	Others	56	12.1%		
Education	No formal education	8	1.7%		
	Primary	16	3.4%		
	Junior high school	77	16.6%		
	Senior high school	147	31.7%		
	Tertiary	216	46.6%		
Employment status	Employed	261	56.3%		
	Unemployed	203	43.8%		
Religion	Christianity	422	90.9%		
	Islam	41	8.8%		
	Others	1	0.2%		

SD, standard deviation.

### Prevalence of PCC utilisation

**Figure 1:**
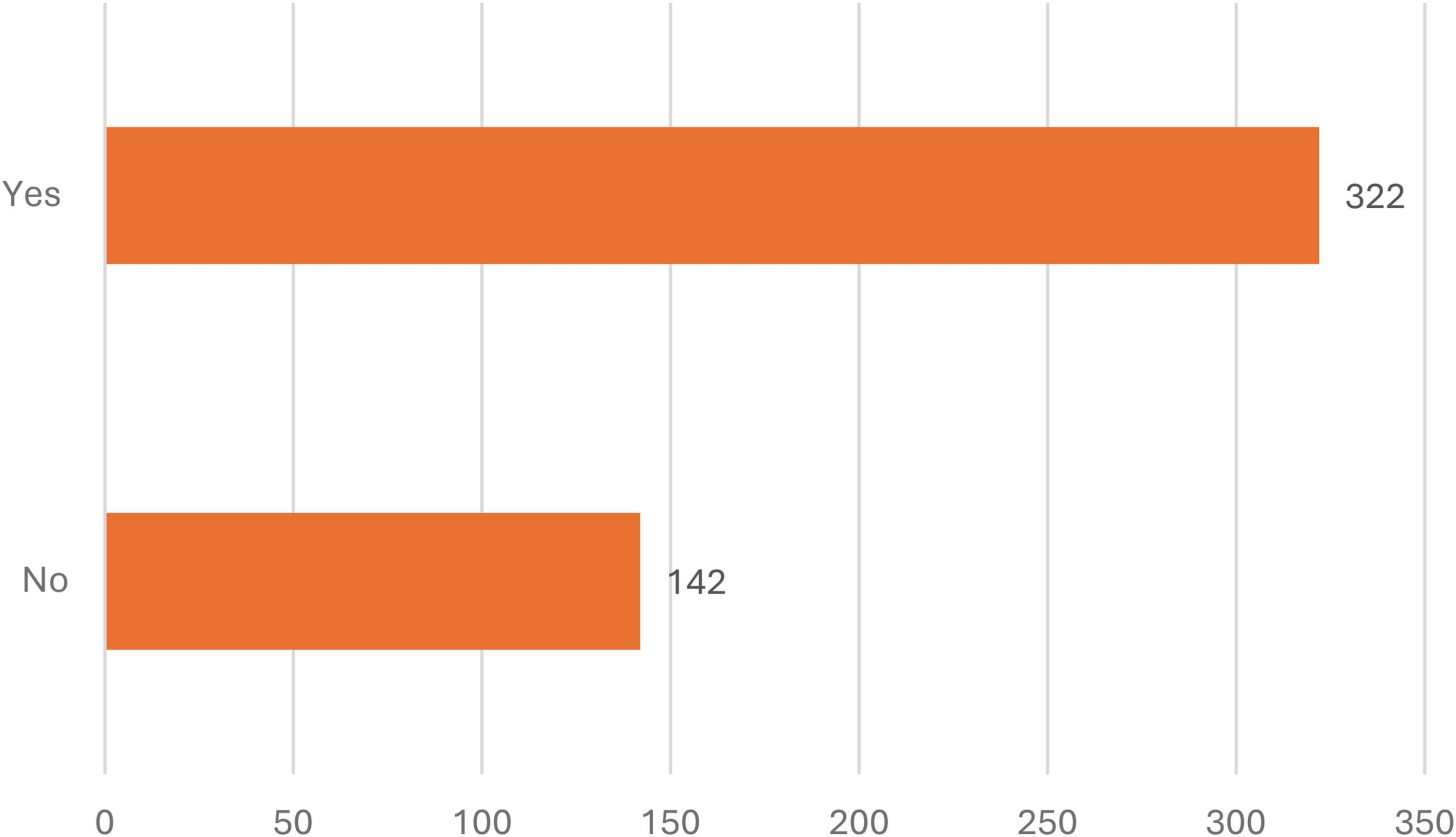
Prevalence of PCC utilisation.

The data indicate that 69.4% reported having received at least one form of PCC service, while 30.6% had never accessed any PCC service (Figure 1).

**Figure 2:**
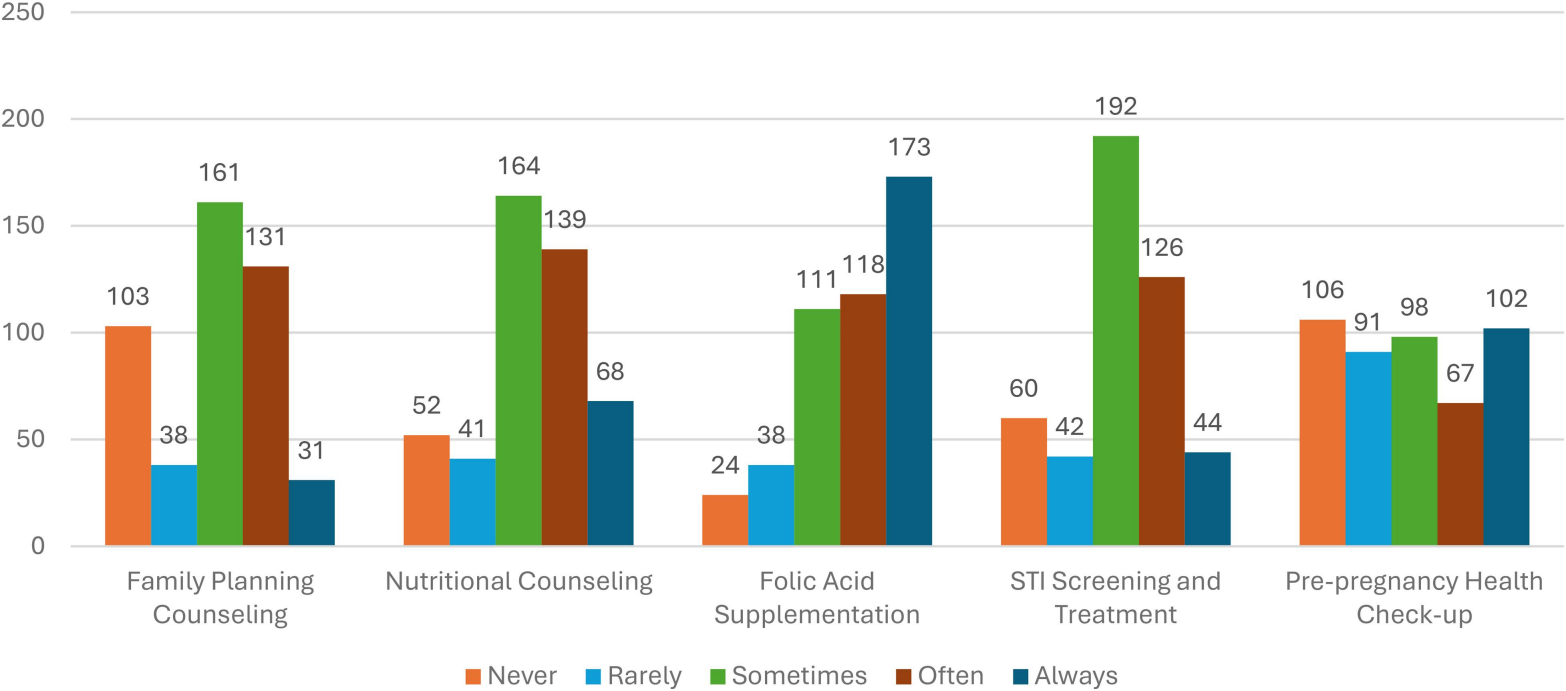
Frequency of access to specific preconception care services among women of reproductive age in the Koforidua Municipality.

The data on the frequency of access to preconception services indicate varying utilisation levels across different service types. Family planning counselling was occasionally accessed, with 34.7% reporting “sometimes” and 28.2% “often”, although 22.2% never received it. Nutritional counselling followed a similar pattern, with 35.3% accessing it “sometimes” and 30.0% “often”, while 11.2% never utilised it. Folic acid supplementation showed the highest regular uptake, as 37.3% reported “always” and 25.4% “often”, suggesting greater awareness and availability. Screening and treatment for sexually transmitted infections were mainly accessed “sometimes” (41.4%), yet 22.0% never or rarely utilised them, indicating service gaps. Pre-pregnancy health check-ups were the least consistent, with 22.8% never and 19.6% rarely attending, while only 22.0% always accessed them (Figure 2).

### Determinant of PCC utilisation among women of childbearing age

Table 2 presents the chi-square analysis examining the association between the independent variables and the level of PCC utilisation. Among the predisposing factors, marital status (*χ*^2^ = 18.6, *p* < 0.001), ethnicity (*χ*^2^ = 10.1, *p* = 0.039), employment status (*χ*^2^ = 16.3, *p* < 0.001), and household size (*χ*^2^ = 8.99, *p* = 0.003) were significantly associated with utilisation For the need factors, previous pregnancy history (*χ*^2^ = 6.44, *p* = 0.011) and counselling from health professionals (*χ*^2^ = 55.4, *p* < 0.001) showed significant relationships. Regarding enabling factors, possession of health insurance (*χ*^2^ = 4.53, *p* = 0.033), daily facility operation (*χ*^2^ = 5.13, *p* = 0.024), free or insured PCC services (*χ*^2^ = 42.3, *p* < 0.001), shorter distance to health facilities (*χ*^2^ = 6.75, *p* = 0.034), and exposure to local radio campaigns (*χ*^2^ = 31.8, *p* < 0.001) were all significantly associated with higher utilisation levels.

**Table 2:** Chi-square test of association between independent variables and the level of PCC utilisation.

		PCC utilisation					
		No		Yes			
Variable	Category	Frequency (*N*)	Percentage (%)	Frequency (*N*)	Percentage (%)	*χ* ^2^	*p*-value
Predisposing factors							
Age	Less than 20 years	12	2.6%	23	5.0%	6.14	0.408
	21–25 years	30	6.5%	46	9.9%		
	26–30 years	43	9.3%	100	21.6%		
	31–35 years	31	6.7%	71	15.3%		
	36–40 years	15	3.2%	55	11.9%		
	41–45 years	9	1.9%	20	4.3%		
	More than 46 years	2	0.4%	7	1.5%		
Marital status	Divorced	0	0.0%	7	1.5%	18.6	<0.001
	Married	51	11.0%	157	33.8%		
	Single	87	18.8%	158	34.1%		
	Widowed	4	0.9%	0	0.0%		
Ethnicity	Akan	67	14.4%	183	39.4%	10.1	0.039
	Dagomba	7	1.5%	13	2.8%		
	Ewe	27	5.8%	64	13.8%		
	Ga	14	3.0%	33	7.1%		
	Others	27	5.8%	29	6.3%		
Education	Junior high school	27	5.8%	50	10.8%	2.71	0.608
	No formal education	2	0.4%	6	1.3%		
	Primary	7	1.5%	9	1.9%		
	Senior high school	45	9.7%	102	22.0%		
	Tertiary	61	13.1%	155	33.4%		
Employment status	Employed	60	12.9%	201	43.3%	16.3	<0.001
	Unemployed	82	17.7%	121	26.1%		
Religion	Christianity	127	27.4%	295	63.6%	1.18	0.553
	Islam	15	3.2%	26	5.6%		
	None	0	0.0%	1	0.2%		
Household size	Less than 5	107	23.1%	279	60.1%	8.99	0.003
	More than 5	35	7.5%	43	9.3%		
Need factors							
Ever been pregnant	No	68	14.7%	114	24.6%	6.44	0.011
	Yes	74	15.9%	208	44.8%		
Attend ANC in your previous pregnancy(s)	No	4	1.4%	6	2.1%	1.01	0.314
	Yes	70	24.8%	202	71.6%		
Experienced any complications in previous pregnancies	No	52	18.6%	140	50.0%	0.135	0.714
	Yes	22	7.9%	66	23.6%		
Ever used any method of contraception	No	57	12.3%	105	22.6%	2.46	0.117
	Yes	85	18.3%	217	46.8%		
Aware of any reproductive health conditions that can affect future pregnancies	No	101	21.8%	200	43.1%	3.51	0.061
	Yes	41	8.8%	122	26.3%		
Received any counseling from a health worker about planning for pregnancy	No	83	17.9%	74	15.9%	55.4	<0.001
	Yes	59	12.7%	248	53.4%		
Enabling factors							
Household Monthly Income (GH₵)	Less than 1,000	80	17.2%	172	37.1%	6.83	0.234
	1,001–2,000	12	2.6%	16	3.4%		
	2,001–3,000	24	5.2%	77	16.6%		
	3,001–4,000	16	3.4%	28	6.0%		
	4,001–5,000	2	0.4%	12	2.6%		
	More than 5,000	8	1.7%	17	3.7%		
Have health insurance	No	10	2.2%	9	1.9%	4.53	0.033
	Yes	132	28.4%	313	67.5%		
The facility operates every day	No	28	6.0%	96	20.7%	5.13	0.024
	Yes	114	24.6%	226	48.7%		
PCC services are provided with adequate privacy and confidentiality	No	16	3.4%	22	4.7%	2.58	0.108
	Yes	126	27.2%	300	64.7%		
PCC services are offered free of charge at the facility	Covered under NHIS	28	6.0%	80	17.2%	42.3	<0.001
	I don’t know	66	14.2%	59	12.7%		
	No	24	5.2%	113	24.4%		
	Yes	24	5.2%	70	15.1%		
Distance to the facility	30 min to 1 h	44	9.5%	141	30.4%	6.75	0.034
	Less than 30 min	79	17.0%	145	31.3%		
	Over 1 h	19	4.1%	36	7.8%		
Local radio campaigns providing information on PCC	No	86	18.5%	105	22.6%	31.8	<0.001
	Yes	56	12.1%	217	46.8%		

### Hierarchical binary logistic regression of association between independent variables and the level of PCC utilisation

Table 3 presents the hierarchical binary logistic regression results showing factors influencing PCC utilisation among women of childbearing age. The overall model demonstrated strong predictive power [*χ*^2^ (35) = 104.3, *p* < 0.001; Nagelkerke R^2^ = 0.470]. Age was a consistent negative predictor, with women aged 21–25 (AOR = 0.03; 95% CI: 0.00–0.63; *p* = 0.024), 26–30 (AOR = 0.02; 95% CI: 0.00–0.45; *p* = 0.013), 31–35 (AOR = 0.01; 95% CI: 0.00–0.19; *p* = 0.002), and 36–40 (AOR = 0.01; 95% CI: 0.00–0.27; *p* = 0.005) significantly less likely to utilise PCC than those under 20 years. Ethnicity showed strong associations, as Akan (AOR = 6.94; 95% CI: 2.47–19.52; *p* < 0.001), Ewe (AOR = 5.65; 95% CI: 1.61–19.86; *p* = 0.007), and Ga (AOR = 5.10; 95% CI: 1.02–25.61; *p* = 0.048) women were substantially more likely to use PCC than other ethnic groups. Education influenced utilisation, with women without formal education being less likely to use PCC (AOR = 0.09; 95% CI: 0.01–0.99; *p* = 0.049). Receiving counselling from a health worker was a strong predictor (AOR = 5.15; 95% CI: 2.30–11.53; *p* < 0.001), and women exposed to local radio campaigns had significantly higher odds of utilisation (AOR = 3.30; 95% CI: 1.49–7.31; *p* = 0.003). Conversely, women uncertain about whether PCC services were covered under the National Health Insurance Scheme were less likely to utilise them (AOR = 0.30; 95% CI: 0.10–0.93; *p* = 0.037).

**Table 3:** Hierarchical binary logistic regression factors influencing PCC utilisation among women of childbearing age in the Koforidua Municipality.

		Model 1		Model 2		Model 3	
Variables	Sub-category	AOR (95% CI)	*P*-value	AOR (95% CI)	*P*-value	AOR (95% CI)	*P*-value
Predisposing factors							
Age (Years)							
Less than 20 years	Ref	Ref	Ref	Ref	Ref	Ref	Ref
	21–25 years	0.18 (0.01, 2.30)	0.188	0.06 (0.00, 1.00)	0.050	0.03 (0.00, 0.63)	0.024
	26–30 years	0.17 (0.02, 1.76)	0.136	0.06 (0.00, 0.83)	0.035	0.02 (0.00, 0.45)	0.013
	31–35 years	0.06 (0.01, 0.63)	0.019	0.02 (0.00, 0.26)	0.003	0.01 (0.00, 0.19)	0.002
	36–40 years	0.10 (0.01, 1.10)	0.060	0.03 (0.00, 0.37)	0.007	0.01 (0.00, 0.27)	0.005
	41–45 years	0.10 (0.01, 1.19)	0.068	0.04 (0.00, 0.61)	0.020	0.02 (0.00, 0.53)	0.018
	More than 46 years	0.15 (0.01, 2.63)	0.195	0.06 (0.00, 1.42)	0.080	0.05 (0.00, 2.12)	0.116
Ethnicity							
Others	Ref	Ref	Ref	Ref	Ref	Ref	Ref
	Akan	4.32 (1.93, 9.65)	<.001	6.55 (2.65, 16.21)	<.001	6.94 (2.47, 19.52)	<.001
	Dagomba	1.92 (0.50, 7.42)	0.343	3.23 (0.69, 15.13)	0.138	2.89 (0.51, 16.27)	0.228
	Ewe	3.26 (1.24, 8.58)	0.017	4.94 (1.67, 14.64)	0.004	5.65 (1.61, 19.86)	0.007
	Ga	3.31 (0.97, 11.33)	0.057	5.37 (1.34, 21.49)	0.018	5.10 (1.02, 25.61)	0.048
Education							
Junior high school	Ref	Ref	Ref	Ref	Ref	Ref	Ref
	No formal education	0.34 (0.05, 2.50)	0.291	0.27 (0.03, 2.12)	0.211	0.09 (0.01, 0.99)	0.049
	Primary	0.38 (0.09, 1.60)	0.187	0.37 (0.08, 1.67)	0.194	0.26 (0.05, 1.47)	0.127
	Senior high school	0.98 (0.44, 2.18)	0.964	1.36 (0.56, 3.27)	0.496	1.18 (0.43, 3.26)	0.745
	Tertiary	1.07 (0.46, 2.51)	0.878	1.50 (0.57, 3.93)	0.411	1.82 (0.55, 6.00)	0.328
Employment Status							
Employed	Ref	Ref	Ref	Ref	Ref	Ref	Ref
	Unemployed	0.48 (0.24, 0.93)	0.030	0.65 (0.30, 1.39)	0.265	0.52 (0.20, 1.32)	0.169
Household size							
Less than 5	Ref	Ref	Ref	Ref	Ref	Ref	Ref
	More than 5	0.57 (0.24, 1.37)	0.209	0.44 (0.17, 1.14)	0.092	0.66 (0.21, 2.09)	0.480
Attend ANC in your previous pregnancy(ies)?							
	No			Ref	Ref	Ref	Ref
	Yes	–	–	1.67 (0.32, 8.78)	0.543	1.46 (0.16, 13.26)	0.738
Experienced any complications in previous pregnancies							
	No			Ref	Ref	Ref	Ref
	Yes	–	–	0.87 (0.42, 1.79)	0.699	0.77 (0.34, 1.72)	0.522
Ever used any method of contraception							
	No			Ref	Ref	Ref	
	Yes	–	–	0.47 (0.22, 0.99)	0.048	0.51 (0.22, 1.21)	0.126
Aware of any reproductive health conditions that can affect future pregnancies							
	No			Ref	Ref	Ref	Ref
	Yes	–	–	1.02 (0.51, 2.05)	0.958	1.06 (0.48, 2.34)	0.881
Received any counseling from a health worker about planning for pregnancy							
	No			Ref	Ref	Ref	Ref
	Yes	–	–	7.72 (3.81, 15.64)	< .001	5.15 (2.30, 11.53)	< .001
Household monthly income (GH₡)							
	Less than 1,000					Ref	Ref
	1,001–2,000	–	–	–	–	0.70 (0.14, 3.55)	0.669
	2,001–3,000	–	–	–	–	0.63 (0.23, 1.78)	0.385
	3,001–4,000	–	–	–	–	0.49 (0.13, 1.90)	0.301
	4,001–5,000	–	–	–	–	1.26 (0.10, 15.33)	0.858
	More than 5,000	–	–	–	–	0.29 (0.06, 1.48)	0.136
Have health insurance							
	No					Ref	refq
	Yes	–	–	–	–	0.31 (0.01, 7.25)	0.465
The facility operates every day.							
	No				Ref	Ref	Ref
	Yes	–	–	–	–	0.52 (0.20, 1.35)	0.180
PCC services are provided with adequate privacy and confidentiality							
	No					Ref	Ref
	Yes	–	–	–	–	0.58 (0.17, 1.98)	0.385
Are PCC services offered free of charge at your facility?							
	No					Ref	Ref
	Covered under NHIS	–	–	–	–	1.03 (0.35,3.00)	0.956
	I don't know	–	–	–	–	0.30 (0.10,0.93)	0.037
	Yes	–	–	–	–	1.33 (0.40,4.51)	0.642
Distance to facility							
	30 min to 1 h					Ref	Ref
	Less than 30 min	–	–	–	–	0.66 (0.29, 1.50)	0.323
	Over 1 h	–	–	–	–	1.03 (0.24, 4.38)	0.966
Heard any local radio campaigns providing information on PCC							
	No					Ref	Ref
	Yes	–	–	–	–	3.30 (1.49, 7.31)	0.003
**Overall Model Test**		**X^2^ (16) = 34.6, *p* = 0.005**		**X^2^ (21) = 38.0, *p* < 0.001**		**X^2^ (35) = 104.3, *p* < 0.001**	
**Nagelkerke's R^2^N**		**0.170**		**0.335**		**0.470**	

AOR, adjusted odds ratio; CI, confidence interval; Ref, reference variable.

## Discussion

This study examined determinants of PCC utilisation among women of reproductive age in Ghana, applying Andersen's Behavioural Model. The findings indicate that 30.6% of women had never accessed any PCC service, while 69.4% of women had received at least one form of PCC. Utilisation varied across service types, with high uptake of folic acid supplementation but low engagement in pre-pregnancy health check-ups. Chi-square results showed that marital status, ethnicity, employment, and household size significantly influenced utilisation. Prior pregnancy experience, counselling from health workers, and access to insured or free PCC also increased use. Logistic regression revealed strong predictive power [*χ*^2^ (35) = 104.3, *p* < 0.001], with age, education, ethnicity, and exposure to radio campaigns emerging as key determinants. Younger, educated, and informed women, particularly Akan, Ewe, and Ga, had higher odds of utilisation. Overall, the findings highlight how socioeconomic, informational, and systemic factors jointly shape PCC uptake in the Koforidua Municipality.

The present study found that 69.4% of women had received at least one form of PCC, while 30.6% had never accessed any service, suggesting moderate utilisation. This proportion is higher than that reported in many sub-Saharan African studies. For instance, Girma et al. (6) in Ethiopia reported a 40% utilisation rate, while Makori (36) recorded 21.6% in Kenya. Similarly, Morema et al. (37) in Kenya observed a 39% rate, and Adjei et al. (23) in Ghana found a 21.4% utilisation rate. These differences may reflect localised health promotion efforts, improved maternal health education, and increased awareness of PCC benefits within the study area. Comparatively, global utilisation varies widely, with higher rates in developed countries such as China (42.2%) (22), while lower rates are noted in developing countries such as Ethiopia (13.4%) (34) and Kenya (21.6%) (38). Studies in Ethiopia have shown similar disparities, ranging from 6.4% in the southern part (39) to 23.4% in Nigeria (40). These variations across and within countries are influenced by policy implementation, accessibility of structured PCC units, socio-cultural beliefs, and the extent of integration of PCC into existing reproductive health programmes. The relatively higher utilisation found in this study, therefore, reflects gradual progress in PCC awareness and accessibility, yet underscores the persistent need for comprehensive, structured PCC services to sustain and improve utilisation.

Findings from this study revealed that age was a significant determinant of PCC utilisation. Women aged 21–25, 26–30, 31–35, and 36–40 years were considerably less likely to utilise PCC services compared with those below 20 years. This means that as age increases, the odds of using PCC decline, suggesting that older women are less likely to seek or use health services before conception. This pattern aligns with reports from Ethiopia and China, where younger women demonstrated greater engagement with PCC interventions (22, 34). Conversely, a study in Jordan (41) found higher PCC awareness among younger women, attributed to improved access to digital health information and school-based health education. These suggest that contextual factors such as education level, health communication channels, and cultural perceptions play a significant role in shaping PCC awareness and practices among women of reproductive age. The reduced likelihood of utilisation among older women may reflect complacency arising from previous childbirth experiences or limited exposure to targeted reproductive health education. These findings highlight the need for age-responsive health promotion programmes that enhance awareness and encourage PCC participation across all reproductive-age groups. Multiparous and older women may underestimate their risk of adverse pregnancy outcomes due to prior experience with childbirth, reducing their motivation to seek preventive care. Moreover, PCC services may not be routinely integrated into reproductive health programmes for these groups, further limiting their engagement. The findings align with Andersen's Behavioural Model of Health Service Utilisation (ABMHSU), where age operates as a predisposing factor influencing individual healthcare-seeking behaviour.

Again, ethnicity was also found to be a strong predictor of PCC utilisation. Women belonging to the Akan, Ewe, and Ga ethnic groups were significantly more likely to access PCC services than women from other ethnic backgrounds. This result is consistent with evidence from Nigeria indicating that socio-cultural orientation and community norms substantially shape maternal health-seeking behaviours (42, 43). Differences in cultural beliefs, access to healthcare information, and community support structures may account for these variations. Promoting culturally tailored reproductive health education and community-specific outreach programmes could help reduce ethnic disparities in PCC use. This correlates with ABMHSU, in which ethnicity functions as a predisposing determinant that affects patterns of healthcare access and utilisation.

In addition, educational attainment was another key factor influencing PCC utilisation. Women without formal education were substantially less likely to access PCC compared with their educated counterparts. Similar associations have been documented, which established that education enhances health literacy and awareness of preventive reproductive health services (44). Educated women are better positioned to interpret health information and make informed decisions about their reproductive well-being. Therefore, expanding educational and health literacy initiatives for women with limited formal education could strengthen equitable access to PCC. This agrees with ABMHSU, where education is identified as a predisposing factor shaping knowledge, perceptions, and engagement with health services.

Further, health communication and system-related factors also demonstrated significant influence. Women who received counselling from healthcare professionals showed higher odds of PCC utilisation, while exposure to local radio messages was also significantly associated with greater utilisation. These findings corroborate studies, which emphasised the importance of interpersonal counselling and mass media campaigns in enhancing PCC uptake (45, 46). Conversely, uncertainty about National Health Insurance Scheme coverage deterred service use, suggesting that financial clarity plays a crucial role in health-seeking decisions. This shows that interpersonal, provider-delivered information is a decisive enabling factor that enhances awareness, motivation, and perceived benefits of preconception care. Strengthening provider-led counselling, expanding media advocacy, and integrating PCC into health insurance packages may be associated with improved service utilisation and could support efforts to address preventable adverse pregnancy outcomes. Collectively, the results highlight that both individual characteristics and contextual influences shape women's likelihood of using PCC, emphasising the need for age- and culture-sensitive counselling and community-based communication strategies to enhance equitable service uptake.

## Implications for policy, practice, and research

The findings reveal notable disparities in PCC utilisation, influenced by sociodemographic and health-system factors. These results underscore the need for targeted policy reforms to integrate PCC into the primary healthcare framework and maternal health programmes. The Ghana Health Service (GHS), Ministry of Health (MoH), and National Health Insurance Authority (NHIA) should prioritise inclusion of comprehensive PCC packages under the NHIS. Additionally, age-specific counselling strategies are needed within primary healthcare and maternal health programmes to ensure equitable access and awareness for all reproductive-age groups. This will ensure financial accessibility, particularly for low-income and rural women. Additionally, local government and district health directorates should collaborate with traditional and community leaders to promote culturally sensitive PCC awareness campaigns in dominant ethnic groups, especially where utilisation remains low. Furthermore, national health promotion units must strengthen media partnerships, particularly with community radio stations, given the strong association between radio exposure and PCC use. Establishing a national preconception policy guideline will promote standardised practice, enhance coordination, and ensure accountability across healthcare levels.

The findings highlight the need for continuous professional education among nurses, midwives, and community health practitioners to strengthen PCC delivery competencies. Health facilities should institutionalise pre-pregnancy health check-ups as part of routine reproductive health services. Broaden PCC messaging beyond first-time mothers to include multiparous and older women, emphasising that advancing maternal age increases risks such as infertility and chromosomal abnormalities. In addition, counselling services should be expanded, as health-worker guidance has been shown to significantly enhance PCC utilisation. Midwives and public health nurses should also intensify family planning, nutritional, and folic acid supplementation education during community outreaches and child welfare clinics. Facility managers should extend service hours and improve accessibility to encourage consistent use. Additionally, the integration of PCC indicators into routine health information systems can improve monitoring and service evaluation. Partnerships between the Ghana Registered Nurses and Midwives Association (GRNMA), reproductive health units, and civil society organisations can support advocacy, training, and dissemination of PCC guidelines.

The study demonstrates the multifactorial determinants of PCC use, highlighting the need for longitudinal and qualitative research to explore contextual and behavioural factors influencing service uptake. Further investigations should assess the effectiveness of NHIS inclusion, radio-based campaigns, and community-driven interventions on sustained PCC utilisation. Comparative studies between rural and urban populations could provide deeper insights into structural barriers. Additionally, implementation research should evaluate the feasibility and outcomes of integrating PCC into reproductive health services within district hospitals and community health planning and services (CHPS) compounds. Future studies should explore motivational and perceptual barriers among older women, while midwives and nurses should incorporate risk communication addressing misconceptions.

## Limitations of the study

This study has several limitations. Its cross-sectional design restricts the ability to establish causal relationships between the determinants and utilisation of PCC. The associations observed should therefore be interpreted as correlations rather than evidence of causation. The use of self-reported data introduces the possibility of recall and social desirability bias, as participants may have over- or under-reported their utilisation of PCC services and related experiences. Moreover, the study was conducted solely within the Koforidua Municipality, which may limit the generalisability of the findings to other settings in Ghana with distinct socio-cultural and healthcare contexts. While Andersen's Behavioural Model provided a robust analytical framework, contextual factors such as partner influence, community norms, and healthcare system constraints were not comprehensively examined. Despite measures to ensure translation accuracy and data quality, potential interviewer bias cannot be entirely excluded. Future research employing longitudinal or mixed methods approaches and broader geographical coverage is recommended to validate and extend these findings.

## Conclusion

This study found that PCC utilisation was moderate and influenced by multiple factors under ABM. Younger, educated women and those of Akan, Ewe, and Ga ethnicity were more likely to use PCC, while counselling from health workers and exposure to local radio campaigns were significantly associated with PCC utilisation. Conversely, uncertainty about coverage under the National Health Insurance Scheme hindered service use. To enhance PCC uptake, it is essential to integrate PCC into national reproductive health programmes, ensure financial inclusion through NHIS, and strengthen culturally appropriate awareness and counselling initiatives. The findings contribute to Sustainable Development Goal 3, target 3.7, by promoting equitable access to sexual and reproductive health services. Improving PCC uptake will prevent avoidable maternal and neonatal complications, enhance pregnancy outcomes, and support Ghana's progress towards sustainable maternal and child health.

## Data Availability

The datasets presented in this study can be found in online repositories. The names of the repository/repositories and accession number(s) can be found below: https://doi.org/10.17605/OSF.IO/CGQT6.
